# Using AGREE II reporting checklist to evaluate the quality of Tuina clinical practice guidelines

**DOI:** 10.3389/fmed.2023.961886

**Published:** 2023-04-18

**Authors:** Fan Huang, Yue Zhang, Chuyu Huang, Mingwang Qiu, Siyi Zhao, Junquan Liang, Zhiyong Fan, Shan Wu

**Affiliations:** ^1^The Second School of Clinical Medicine, Guangzhou University of Chinese Medicine, Guangzhou, Guangdong, China; ^2^Clinical Medical College of Acupuncture Moxibustion and Rehabilitation, Guangzhou University of Chinese Medicine, Guangzhou, Guangdong, China; ^3^Shenzhen Bao'an Chinese Medicine Hospital, The Seventh Clinical Medical School of Guangzhou University of Chinese Medicine, Shenzhen, Guangdong, China; ^4^The Brain Cognition and Brain Disease Institute (BCBDI), Shenzhen Institute of Advanced Technology, Chinese Academy of Sciences (CAS), Shenzhen-Hong Kong Institute of Brain Science-Shenzhen Fundamental Research Institutions, Shenzhen, Guangdong, China; ^5^University of Chinese Academy of Sciences, Beijing, China

**Keywords:** traditional Chinese medicine, Tuina, evaluation II instrument, guidelines, AGREE II instrument

## Abstract

**Objective:**

The objective of this study is to evaluate the methodological quality of Tuina clinical practice guidelines (CPGs).

**Methods:**

Computer searches of China National Knowledge Infrastructure (CNKI), Chinese Technical Periodicals (VIP), Wanfang Data Knowledge Service Platform, PubMed, Cochrane Library, Embase, and other databases were conducted to search for published guidelines on Tuina, with a search time frame from database creation to March 2021. Four evaluators independently used the Appraisal of Guidelines for Research and Evaluation II instrument to evaluate the quality of the included guidelines.

**Results:**

A total of eight guidelines related to Tuina were included in this study. The quality of reporting was low in all included guidelines. The highest quality report had a total score of 404 and was rated as “highly recommended.” The worst guideline had a final score of 241 and was rated as “not recommended.” Overall, 25% of the included guidelines were recommended for clinical use, 37.5% were recommended after revision, and 37.5% were not recommended.

**Conclusion:**

The number of existing Tuina clinical practice guidelines is limited. The methodological quality is low, far from the internationally accepted clinical practice guideline development and reporting norms. In the future, reporting specifications of guidelines and the methodology of guideline development, including the rigor of the guideline development process, the clarity, application, and independence of reporting, should be emphasized in the development of the Tuina guidelines. These initiatives could improve the quality and applicability of clinical practice guidelines to guide and standardize the clinical practice of Tuina.

## Advantages and limitations of this study

- To our knowledge, no studies have assessed the quality of Tuina clinical practice guidelines using the AGREE II checklist.
- The included guidelines were measured using the AGREE II instrument, which allows for the evaluation of guideline development methodology.
- A total of eight relevant guidelines were included in this study, which involved targeting different populations.
- The study showed inadequate reporting quality in some areas.
- The study suggests that AGREE II may be somewhat challenging in helping determine the specificity of recommendations related to traditional Chinese medicine or traditional Chinese medicine techniques.

## 1. Introduction

Tuina is a non-pharmacological therapy (1). At present, many studies have demonstrated that Tuina is effective in treating neck pain, shoulder pain, low back pain, and other pain caused by spinal cord disease (2–5). According to the 2016 guidelines for orthopedic therapy for non-specific low back pain formulated by the American Orthopedic Association (6), Tuina is recommended as an important treatment method because of its highly scientific nature and safety. Chinese orthopedic rehabilitation experts also believe that Tuina is significantly better than conventional treatment in alleviating the pain of lumbar disc herniation (7).

The guidelines based on the evidence of SR are recommendations that could provide patients with reliable healthcare services after balancing the pros and cons of various interventions and help clinicians make medical decisions. Only high-quality guidelines can effectively fulfill their role in clinical guidance; however, the uneven quality of the current clinical practice guidelines (CPGs) makes it necessary to conduct a quality evaluation of the guidelines in a timely manner (8). As the world's attention to Tuina is increasing, more and more clinical guidelines become available. However, these guidelines have not yet been standardized, and the definition and use of Tuina vary in China and in other countries. In addition, the quality of clinical guidelines in Tuina is uncertain, and the tools currently in use cannot accurately address quality assessment and reporting issues in a single statement.

Appraisal of Guidelines for Research and Evaluation II (AGREE II), which is developed by a group of experts, is used for quality assessment and reporting (9, 10). AGREE II has become an international standard for evaluating the methodological quality and transparency of CPGs, and several organizations have incorporated AGREE as part of their CPG programs (11). Therefore, the quality of guidelines determines whether they are suitable for clinical application and promotion. This study aims to conduct methodological and report quality evaluation by the international guidelines for quality evaluation tool AGREE II to evaluate the published clinical practice guidelines for Tuina. It provides a reference for the formulation and update of future guidelines, to enhance the quality of domestic and international guidelines so as to better play its guiding role.

## 2. Methods

### 2.1. Literature search

Guidelines meeting the eligibility criteria were searched in English and Chinese using a computer program to avoid subjective interpretation. A total of 11 databases, including the National Institute for Health and Care Excellence (NICE), Agency for Healthcare Research and Quality (AHRQ), PubMed, Embase, AMED (Allied and Alternative Medicine), Cumulative Index to Nursing and Allied Health Literature (CINAHL), WanFang Data, China National Knowledge Infrastructure (CNKI), Chinese Technical Periodicals (VIP), and Chinese Biomedical Literature Database (CBM), collection of clinical practice guidelines for Tuina at CHINA and abroad, were searched for articles from inception until March 2021. At the same time, the Google Academic and the Yimaitong databases were searched to supplement the acquisition of relevant guidelines. The search adopts a combination of subject terms and free words. The search terms include “massage” or “Tuina” or “Chinese manual therapy” AND “guideline” or “guidance” or “recommendation” or “consensus” or “policy”.

### 2.2. Selection criteria

We included clinical practice guidelines published by international, national, or regional groups for the application of Tuina. The guidelines were included if they met the following criteria: (1) published in English or Chinese language, (2) peer-review publications, and (3) published between 2000 and 2021, and we also excluded patient-used guidelines, guideline commentaries, guideline interpretations, and translated versions of original guidelines.

### 2.3. Data extraction

Two researchers (Mingwang Qiu and Yue Zhang) used the document management software EndNote X9 to screen the documents and extract data independently. Disagreement between the two parties shall be resolved through a discussion by a third party (Fan Huang).

### 2.4. Quality evaluation

In total, four researchers (Mingwang Qiu, Yue Zhang, Fan Huang, and Siyi Zhao) used AGREE II to evaluate the quality of the inclusion guidelines, including 23 items in 6 areas and 2 overall assessment items. The minimum score for each item was 1 point, and the maximum score was 7 points (9). We have calculated the final score of each field according to the formula as follows: each field score = (actual score-minimum possible score)/(maximum possible score-minimum possible score) × 100% (9).

The AGREE II instrument does not offer guidance on the cutoff scores to determine the quality of each domain; to determine the overall quality and recommendation level, we based our approach on the methods of previous research, guidelines were classified as strongly recommended when the standardized percentages of all six domains were above 60% and recommended when the standardized percentages ranged from 30% to 60% in more than three domains. However, guidelines are not recommended when the standardized percentages were < 30% in more than three domains.

Before the formal evaluation, all investigators were trained, and one guide was independently pre-scored. Then the group discussed and negotiated to ensure that the four evaluators had basically the same understanding of each item and had the same evaluation criteria.

### 2.5. Statistical analysis

Statistical analysis used IBM SPSS Statistics 25 software to calculate the ICC value to verify the consistency of the evaluators when using the evaluation tool. Using Excel 2016 for calculating the scores of the AGREE II tool, the mean and standard deviation of the scores in each field and the proportion of each part were calculated. The agreement between the four reviewers was measured by the intra-group correlation coefficient (ICC) and 95% confidence interval (CI). The degree of agreement between 0.01 and 0.20 was considered minor, the degree of proportionality between 0.21 and 0.40 was considered moderate, the degree of proportionality between 0.41 and 0.60, the substantive degree between 0.61 and 0.80, and the agreement between 0.81 and 1.00 were considered very good. *P* < 0.05 indicates statistical significance. All tests were double-sided. We used SPSS version 25.0 for statistical analysis. The ICC of the scores given by the four evaluators would be analyze.

### 2.6. Patient and public involvement

No patient was involved.

## 3. Results

### 3.1. Selection of studies

**Figure 1:**
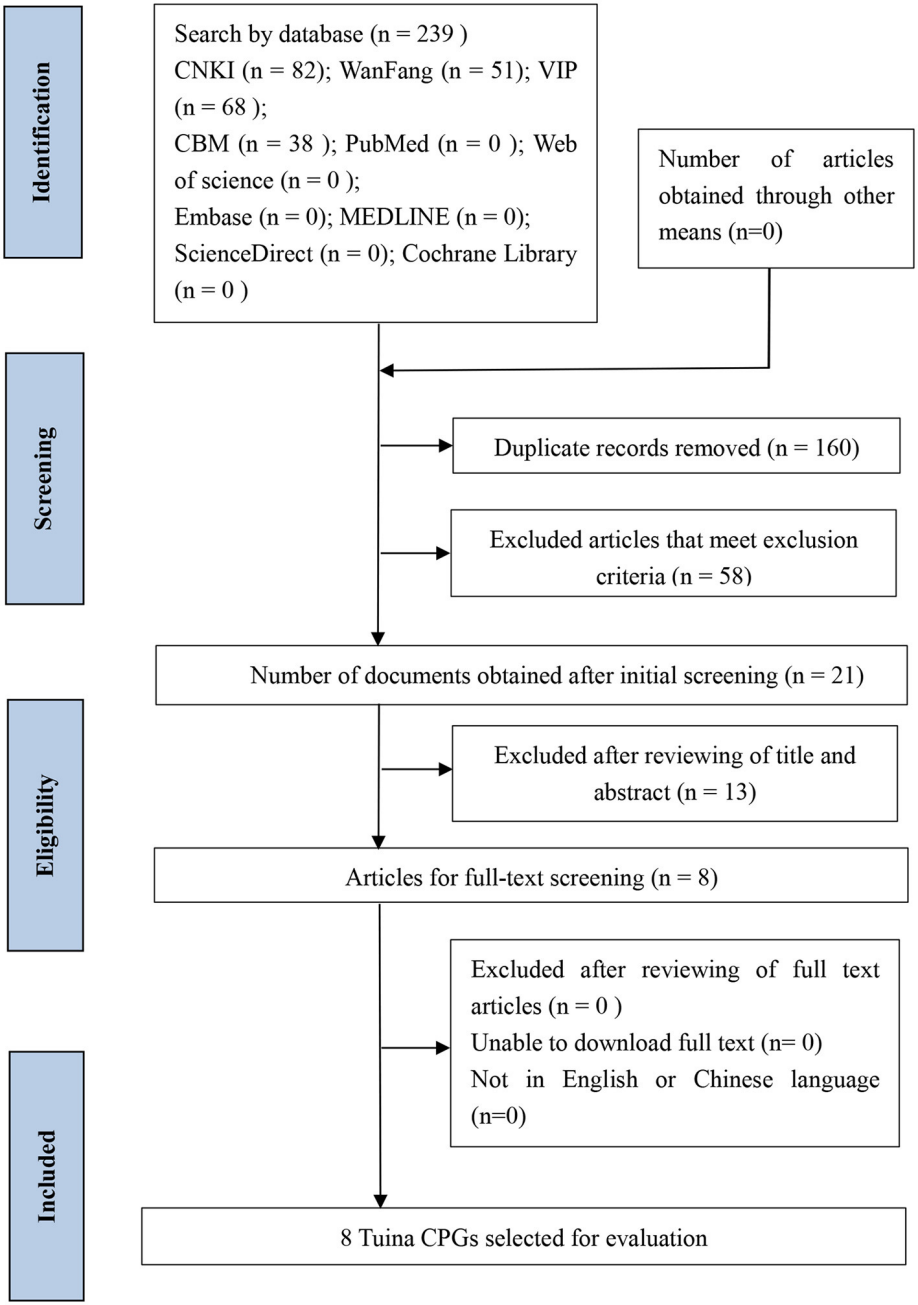
Flowchart of Tuina guidelines searching and selection.

The initial search detected 239 related publications, and EndNote X9 excluded 160 duplicate records. After reading the title and abstract, 13 records were excluded from the preliminary screening. After the full-text screening, a total of eight Tuina guidelines were included (12–19) for SR through AGREE II. The literature search and screening process are shown in Figure 1.

### 3.2. General characteristics

As shown in Table 1, all the guidelines evaluated in this study originated from China and were published from 2010 to 2020. Three of the guidelines (14–16) were published in the same journal in 2010. However, a major shortcoming of the former three articles was that there is no evidence of the source of the guideline writing. In total, seven of the eight guidelines (9, 11–16, 18, 19) were aimed at clinicians or Tuina practitioners specifically. Seven (12–17, 19) guidelines were from different Chinese medicine universities or affiliated hospitals, and the remaining one (18) was from the China HQCC.

**Table 1:** Characteristics of the included guidelines.

**Reference**	**Year of publication**	**Country**	**Organizational affiliation**	**Journal**	**Target**	**Evidence based**
Li (12)	2014	China	NUCM	*PEDIATRICS OF TCM*	Physicians	Investigation
Chen et al. (13)	2017	China	CACM-JPHCM	*PEDIATRICS OF TCM*	Physicians	Expert opinion
Lei et al. (14)	2010	China	CACM-TAFHGUCM	*CATCM*	Physicians, Tuina practitioners	NR
Sun et al. (15)	2010	China	CACM-SYTCMPTS	*CATCM*	Physicians, infantile Tuina practitioners	NR
Shen et al. (16)	2010	China	CACM-TAFHGUCM	*CATCM*	Physicians, Tuina practitioners	NR
Qing et al. (17)	2018	China	DHBUCM	*Int J TCM*	NR	Expert panel
Yan et al. (18)	2020	China	HQCC**-**CPTSCCC-SAHTUTCM	*Tianjin J TCM*	Physicians	Expert panel
Liu (19)	2020	China	BHTCM	*World J Int Trad West Med*	Physicians, infantile Tuina practitioners	Expert panel

NR, not reported; NUCM, Nanjing University of Chinese Medicine; CACM, China Association of Chinese Medicine; JPHCM, Jiangsu Province Hospital of Chinese Medicine; TAFHGUCM, The First Affiliated Hospital of Guangxi University of Chinese Medicine; SYTCMPTS, Shanxi Yuncheng Traditional Chinese Medicine Pediactric Tuina School; DHBUCM, Dongzhimen Hospital Beijing University of Chinese Medicine; HQCC-CPTSCCC, HQCC Chinese Pediactric Tuina of Standardization Construction and Certification Committee; SAHTUTCM, Second Affiliated Hospital of Tianjin University of TCM; BHTC, Beijing Hospital of Traditional Chinese Hospital; TCM, traditional Chinese medicine.

### 3.3. Quality assessment of guidelines and strength of recommendation

Table 2 shows the AGREE II standardization field score for each Tuina CPG and its overall recommendations. The scope and purpose of the field and the clarity and presentation achieved the highest average scores of 70% and 67% (ranged 60–82% and 51–82%, respectively). The average score for stakeholder participation in the domain was 51% (ranged 33–61%), and only one guide scored more than 60%. The largest score range was editorial independence (17–94%). A total of three guides (37.5%) scored 17, and five guides (62.5%) scored <50%. Editorial independence and applicability produced the lowest average scores of 40% and 47% (ranged 17–94% and 21–64%, respectively). Unexpectedly, the four guidelines (26.7%) had the lowest score due to the failure to describe the criteria for selecting evidence and making recommendations clearly.

**Table 2:** AGREE II domain scores of Tuina guidelines and overall assessment.

**Reference**	**Scope and purpose**	**Stakeholder involvement**	**Rigor of development**	**Clarity of presentation**	**Applicability**	**Editorial independence**	**Total score**	**Overall assessment**
Li (12)	60	33	40	56	35	17	241	Not recommended
Chen et al. (13)	74	61	68	51	56	94	404	Strongly recommended
Lei et al. (14)	64	57	59	65	57	17	319	Recommended with modifications
Sun et al. (15)	69	57	52	78	61	17	334	Recommended with modifications
Shen et al. (16)	72	56	61	82	58	19	348	Recommended with modifications
Qing et al. (17)	79	44	59	78	64	77	401	Strongly recommended
Yan et al. (18)	60	46	41	71	21	56	295	Not recommended
Liu (19)	82	57	36	57	21	23	276	Not recommended

In general, the Practical Guidelines for Treating Prophylactic Diseases of Tuina Intervention of Spleen Deficiency (13) in Children's Guide had high scores in all areas and is listed as “strongly recommended” in clinical practice, “recommendation of 3 types of Tuina CPG (37.5%),” and three types (37.5%) “not recommended.” The agreement of the overall reviewer was very good (ICC:0.901, 95% CI).

After four reviewers' SR, we obtained the AGREE II scores and total scores in each field of the eight guides, as shown in Table 3. Chen et al. (13) and Qin et al. (17) served as the first author's guide for a total score of more than 400; therefore, we can strongly recommend using these two Tuina guidelines. The guidelines with Li (12), Yan et al. (18), and Liu (19) as the first authors had a total score of < 300, so we do not recommend using these three guidelines. The guide, led by Lei et al. (14), Sun et al. (15), and Shen et al. (16) as the first author, scored between 300 and 400. These three guides can be recommended after upgrading and improving modifications.

**Table 3:** The intra-group correlation coefficients of the scores.

**Item**	**Domain**	**Description**	**ICC (95% CI)**
1	Scope and Purpose	The overall objective(s) of the guideline is (are) specifically described.	0.885
2		The health question(s) covered by the guideline is (are) specifically described.	0.842
3		The population (patients, public, etc.) to whom the guideline is meant to apply is specifically described.	0.867
4	Skate holder involvement	The guideline development group includes individuals from all relevant professional groups.	0.862
5		The views and preferences of the target population (patients, public, etc.) have been sought.	1.000
6		The target users of the guideline are clearly defined.	0.907
7	Rigor of development	Systematic methods were used to search for evidence.	0.800
8		The criteria for selecting the evidence are clearly described.	0.938
9		The strengths and limitations of the body of evidence are clearly described.	0.813
10		The methods for formulating the recommendations are clearly described.	0.838
11		The health benefits, side effects, and risks have been considered in formulating the recommendations.	1.000
12		There is an explicit link between the recommendations and the supporting evidence.	0.891
13		The guideline has been externally reviewed by experts prior to its publication.	0.862
14		A procedure for updating the guideline is provided.	0.932
15	Clarity of presentation	The recommendations are specific and unambiguous.	0.828
16		The different options for management of the condition or health issue are clearly presented.	0.806
17		Key recommendations are easily identifiable.	0.886
18	Applicability	The guideline describes facilitators and barriers to its application.	0.887
19		The guideline provides advice and/or tools on how the recommendations can be put into practice.	0.865
20		The potential resource implications of applying the recommendations have been considered.	0.870
21		The guideline presents monitoring and/or auditing criteria.	0.800
22	Editor independence	The views of the funding body have not influenced the content of the guideline.	0.956
23		Competing interests of guideline development group members have been recorded and addressed.	0.938

The agreement between the four reviewers was measured by the intra-group correlation coefficient (ICC) and 95% confidence interval (CI). The degree of agreement between 0.01 and 0.20 was considered minor; the degree of proportionality between 0.21 and 0.40 was moderate; and the degree of proportionality between 0.41 and 0.60, the substantive degree between 0.61 and 0.80, and the agreement between 0.81 and 1.00 were very good. *P* < 0.05 indicates statistical significance. All tests were double-sided. We used SPSS version 25.0 for statistical analysis. By analyzing the intra-group correlation coefficients of the scores given by the four evaluators, we can see that the ICC value of each field was >0.81, as shown in Table 3. It can be considered that the scores given by the evaluators within the group were highly consistent.

### 3.4. Frequency statistics of disease

**Figure 2:**
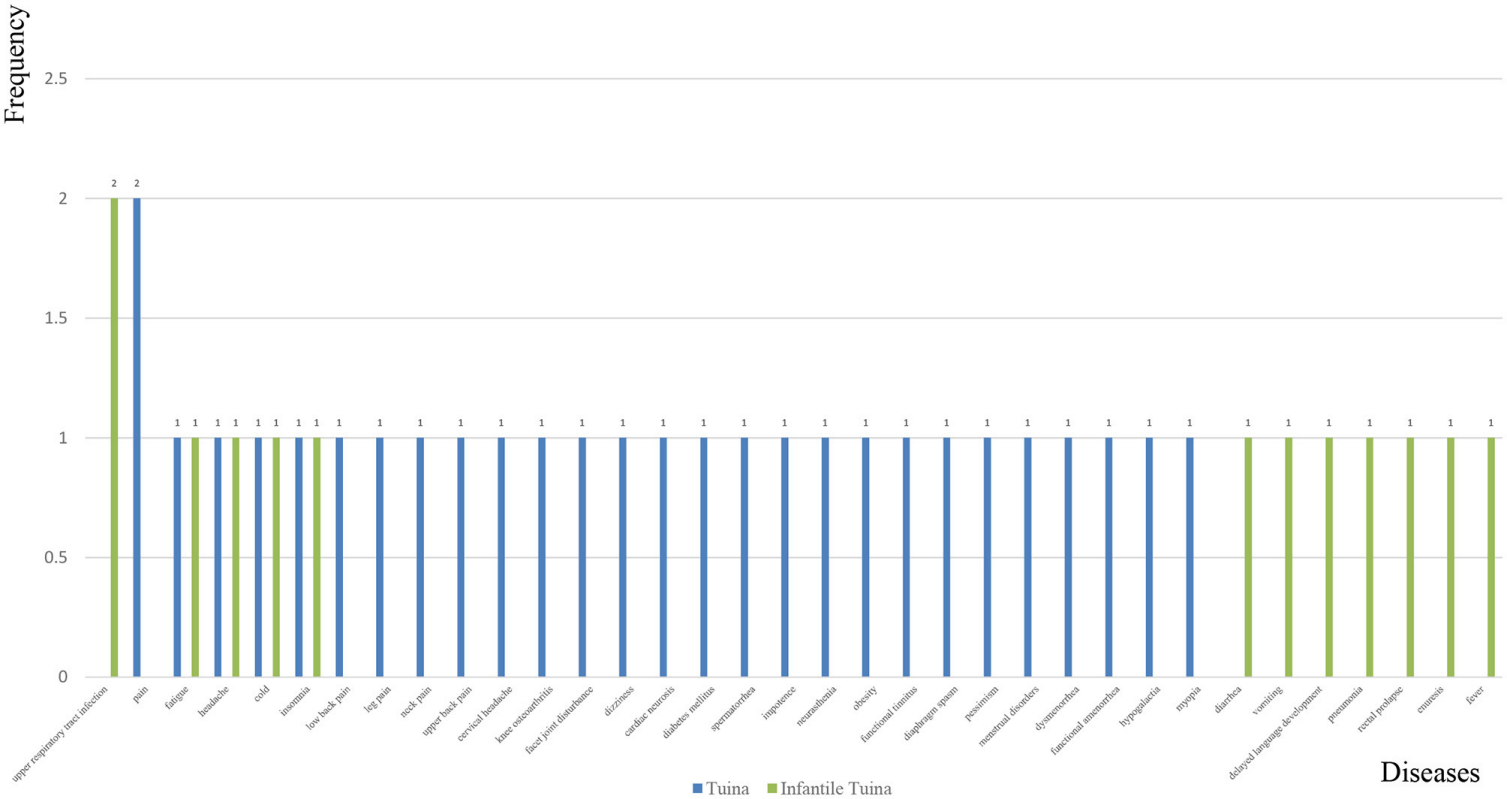
Names of diseases included in the literature (abscissa axis) and their frequency (ordinate axis).

As shown in Figure 2, the abscissa axis is the names of the diseases included in the literature, and the ordinate axis is the frequency with which they appear in the literature. A total of 35 diseases were included; of which, 13 were specific to children.

## 4. Discussion

This study systematically compared the strengths and weaknesses of eight existing guidelines with AGREE II reporting checklist to provide a reference for the development of Tuina CPGs.

The results of this study highlighted the poor applicability of current Tuina CPGs. Meanwhile, unclear articulation of the strengths and weaknesses of the recommendations, the lack of supporting tools, potential resources, and monitoring and auditing criteria may be direct causes of the low scores in this area.

Clinical practice guidelines are defined as systematically developed statements to assist practitioner and patient decisions about appropriate healthcare for specific clinical circumstances (20). As society gradually recognized the therapeutic effect of Tuina, CPGs will be in increasing demand globally. In the present study, systematic research on CPGs for Tuina was performed, and key messages were summarized. We found it difficult to evaluate the individual and community as the available CPGs for Tuina lack reports on obtaining evidence and reaching recommendations.

The implication of this study is that, as a result of our findings, a careful reassessment of the quality standards of existing Tuina guidelines can be called for, and quality improvements can be made in practice to facilitate the development and reporting of more Tuina guidelines in the future. In this study, the quality of massage guidelines was highly heterogeneous across domains. The level of evidence and strength of recommendations also varied widely across the categories of guidelines. With regard to the evidence base for the inclusion of massage guidelines in this study, our findings suggest that many guidelines are based primarily on a low level of evidence or expert opinion, offering a lack of quality evidence and guidelines that do not incorporate evidence.

At present, the quality of Chinese domestic guidelines is generally low, and low-quality guidelines not only fail to guide clinical practice but also may even hinder it. In this study, we chose to use the international guideline quality evaluation tool AGREE II to evaluate the quality of Chinese domestic journals publishing clinical practice guidelines for Tuina in recent years, to monitor the changes in the quality of Tuina clinical guidelines, to provide a reference for the development and updating of guidelines in the future, and to improve the quality of domestic guidelines so that they can better play their guiding role. Compared with the Reporting Items for Practice Guidelines in healthcare (RIGHT) statement completed by the Chinese lead, we use AGREE II to evaluate the guidelines because the international common AGREE II to assess the quality of guidelines, AGREE II has been supported and recognized by many healthcare organizations, which can help Tuina better align with international standards (21). By comparing the original AGREE with the AGREE II program, we chose the latest AGREE II tool for guideline evaluation because the AGREE II program changes the original 12 entries from AGREE to assess guideline quality better as well as provide a methodological strategy for guideline development, informing guideline developers on what to report in the guideline, what information to report, and how to report it (9).

The following part leads to the limitations of our study. First, due to language limitations, this study only collects guidelines written in Chinese and English, which could not include documents from a few countries and regions. Second, the scores of AGREE II are not weighted, so the guideline' recommendation level is only based on the number of fields that meet the standard. There may be cases where the recommended results are not in conformity with the quality of the guideline. Third, the AGREE II score was based on the reports of CPG developers, and low domain scores might be due to poor methodology in the development process of CPGs. Fourth, some guidelines may be in development and thus were not accessible to our research team.

The total quality of CPGs is not high in our country (22), and therefore, how to develop a high-required guideline is an issue that guideline makers need to consider. It is expected that Tuina practitioners can institute high-level CPGs that are suitable for China based on the methodology and normative reports formulated by the guidelines.

## 5. Conclusion

The number of CPGs for Tuina has proliferated in recent years, but their average quality is unsatisfactory, especially in the domain of stakeholder participation, editorial independence, and the description of the criteria for selecting evidence and making recommendations. In the subsequent development of CPGs in Tuina, guideline developers need to further improve guideline methodology and reporting specifications of the domain mentioned earlier. Evaluation tools for guidelines in Tuina and CPGs for Tuina should be developed according to standardized guideline development methods that are consistent with Chinese national conditions. Emphasis should be placed on strengthening the promotion and application of Tuina guidelines.

## Data availability statement

The original contributions presented in the study are included in the article/Supplementary material, further inquiries can be directed to the corresponding authors.

## Author contributions

All authors listed have made a substantial, direct, and intellectual contribution to the work and approved it for publication.
