# Supplementary material for: Using AGREE II reporting checklist to evaluate the quality of Tuina clinical practice guidelines
Source: Front Med (Lausanne). 2023 Apr 18;10:961886. doi: 10.3389/fmed.2023.961886 (PMC10152900; doi:10.3389/fmed.2023.961886)
Supplement: Supplementary file 1 [file Data_Sheet_1.pdf]

CNKI (n = 82);

1.TI：“tuina” OR “zhenggu” OR “anmo” OR “shoufa” OR “zhengji”

2.TI： “zhinan” OR “gongshi” OR “guifan”

3. #1 AND #2

WanFang (n = 51);

1.TI：“tuina” OR “zhenggu” OR “anmo” OR “shoufa” OR “zhengji”

2.TI： “zhinan” OR “gongshi” OR “guifan”

3. #1 AND #2

VIP (n = 68);

1.TI：“tuina” OR “zhenggu” OR “anmo” OR “shoufa” OR “zhengji”

2.TI： “zhinan” OR “gongshi” OR “guifan”

3. #1 AND #2

CBM (n = 38);

1.TI：“tuina” OR “zhenggu” OR “anmo” OR “shoufa” OR “zhengji”

2.TI： “zhinan” OR “gongshi” OR “guifan”

3. #1 AND #2

PubMed (n = 0);

1.MeSH descriptor: [Musculoskeletal Manipulations] explode all trees

2.(Tuina OR Chineses manual therapy OR manipulation OR chiropractic OR rehabilitation):ti,ab

3.#1 OR #2

4. MeSH descriptor: [Guideline] explode all trees

5. (guideline OR guidance OR recommendation OR consensus OR policy):ti,ab

6. #4 OR #5

7. #3 AND #6

Web of science (n = 0);

1.exp Musculoskeletal Manipulations.mp.

2.(Tuina OR Chineses manual therapy OR manipulation OR chiropractic OR rehabilitation) .tw.

3. #1 OR #2

4.exp Guideline.mp.

5. (guideline OR guidance OR recommendation OR consensus OR policy).tw.

6. #4 OR #5

Embase (n = 0);

1.exp Musculoskeletal Manipulations.mp.

2.(Tuina OR Chineses manual therapy OR manipulation OR chiropractic OR rehabilitation) .tw.

3. #1 OR #2

4.exp Guideline.mp.

5. (guideline OR guidance OR recommendation OR consensus OR policy).tw.

6. #4 OR #5

MEDLINE (n = 0);

1.MeSH descriptor: [Musculoskeletal Manipulations] explode all trees

2.(Tuina OR Chineses manual therapy OR manipulation OR chiropractic OR rehabilitation):ti,ab

3.#1 OR #2

4. MeSH descriptor: [Guideline] explode all trees

5. (guideline OR guidance OR recommendation OR consensus OR policy):ti,ab

6. #4 OR #5

7. #3 AND #6

ScienceDirect (n = 0);

Title, abstract, keywords: (guideline OR guidance OR recommendation OR consensus OR policy) AND (Tuina OR Chineses manual therapy OR manipulation OR chiropractic OR rehabilitation)

Cochrane Library (n = 0).

1.MeSH descriptor: [Musculoskeletal Manipulations] explode all trees

2.(Tuina OR manipulation OR chiropractic OR rehabilitation):ti,ab,kw (Word variations have been searched)

3. #1 or #2

4. MeSH descriptor: [Guideline] explode all trees

5. (guideline OR guidance OR recommendation OR consensus OR policy)

6. #4 OR #5

7. # 3 AND #6
